# Patient and professional attitudes towards research in general practice: the RepR qualitative study

**DOI:** 10.1186/1471-2296-15-136

**Published:** 2014-07-21

**Authors:** Jean-Sébastien Cadwallader, Jean-Pierre Lebeau, Evelyne Lasserre, Laurent Letrilliart

**Affiliations:** 1 Department of General Practice, University of Tours, 10 Boulevard Tonnellé, BP 3223, Tours, Cedex 1 37032, France; 2 SIS EA 4129 laboratory, University of Lyon, Lyon, France; 3 Department of General Practice, EES, University of Tours, 10 Boulevard Tonnellé, BP 3223, Tours, Cedex 1 37032, France; 4 Department of Anthropology, University of Lyon, Lyon, France; 5 Department of General Practice, University of Lyon, Lyon, France

**Keywords:** General practice/Family medicine, Research, Qualitative study, Attitudes of Health Personnel

## Abstract

**Background:**

Since the 1990s, professional institutions worldwide have emphasised the need to develop research in general practice to improve the health of the population. The recent creation of professorships in general practice in French Universities should foster research in this field. Our aim was to explore the views of patients and relevant professionals on research in general practice.

**Methods:**

Qualitative study, using the grounded theory approach according to Strauss and Corbin, conducted in 2010 in three French regions. Nine focus groups were run to data saturation, and included 57 participants in four different categories: patients, non-academic GPs, academic GPs, academics in other disciplines.

**Results:**

Most of the participants in the four categories described research in general practice as specific to the population managed and relevant for health care. They considered that its grounding in day-to-day practice enabled pragmatic approaches. The influence of the pharmaceutical industry, rivalries between university disciplines and a possible gap between research and practice were considered as pitfalls. The barriers identified were representations of the medical researcher as a “laboratory worker”, the lack of awareness of any research in the discipline, and lack of time and training. While the views of patients and non-academic GPs are mostly focused on professional issues and the views of academics other than GPs on technical issues, academic GPs are in a position to play a role of interface between the universities and general practices.

**Conclusions:**

Although the role of GPs in research is perceived differently by the various protagonists, research in general practice has an undisputed legitimacy in France. Solutions for overcoming the identified barriers include research networks with appropriate resources and training and scientifically sound collaborative research projects, as already implemented in leading countries.

## Background

A strong primary care sector helps the health system to improve health, reduce mortality from prevalent diseases and reduce health inequalities, regardless of the type of health care organization [1]. Early international initiatives for developing research in general practice emerged in the 1990s, led in particular by Australia and the Netherlands [2]. In 2003, an invitational WONCA conference in Kingston, Canada, raised the question of the need for research in primary medical care to improve health care worldwide [3]. In 2009, the European General Practice Research Network published a research agenda, pointing out evidence gaps and research needs as a basis for planning research [4].

Nevertheless, many countries, including France, have seen little research in general practice, even though most health problems in the population are managed in primary care [5]. This shortfall, described as the “law of inverse opportunity”, results in a shortage of relevant scientific data in general practice [6]. Between 1960 and 2003, a corpus of about 50,000 references retrieved from the 15 international scientific journals of family medicine/general practice only amounted to 1/20th of the volume of references in cardiology [7]. Moreover, most of the publications on general practice research come from very few countries, mainly the UK, the USA and the Netherlands [8].

The recent appointment of professors and lecturers in general practice in French Universities, with a research mandate alongside teaching and primary care practice, should lead to the development of primary care research [9]. A quantitative study previously indicated that nearly one third of French General Practitioners (GPs) were willing to participate in research projects as investigators [10]. While GPs are prepared to take part in research, a deeper and broader analysis of the views of all the stakeholders involved in this process (GPs, academics, patients) is needed. Their views are necessary to find solutions for overcoming the potential barriers and build general practice research capacities.

The aim of the RepR study (Representations of Research) was to explore the views of patients and relevant professionals on research in general practice.

## Methods

We conducted a qualitative study based on focus groups, since the subject matter was open to interpretation. The grounded theory approach according to Strauss and Corbin was used to explore the participants’ views and interactions, in a non-explicit context and without a predefined framework [11].

### Sampling

A purposive sampling procedure was carried out in three large, geographically distant French regions (North, Centre West, Centre East), in order to collect a wide range of opinions. Four different categories of participants were considered: patients (P), non-academic general practitioners (GP), academic GPs (AGP) and academics in other fundamental or clinical disciplines (OA). Academic GPs and other academics were recruited through regional university departments of general practice, non-academic GPs through continuing medical education organisations or training practice, and patients from participating GPs’ practices. Further participants were found using a snowball technique. Patients were recruited according to various socio-economic characteristics (age, gender, profession, family structure, health status), and non-academic GPs according to various practice settings (type of area, number of collaborators, training practice). Academic GPs were GPs having a position as University teachers, with or without research activity. Academics in other disciplines had significant experience in research.

### Data collection

Focus groups were conducted separately for each category of participants, in order to avoid any domination effect among the various participants. A common topic guide was designed for all categories of participants. The first topic guide was elaborated inductively, with the agreement of one participant from each participant category. It was subsequently adjusted on the basis of the issues raised in the first two focus groups The questions were: What is medical research? What is research in general practice? What is the relevance of this research? What are the facilitators and barriers? What setting for this research? What role for GPs in this research? What kind of training for GP research? Nine focus groups were organized throughout France between January and June 2010, two to three for each category of participant, until data saturation was reached at least globally, and not necessarily for each category of participants. To create a reassuring atmosphere, they were conducted in a familiar setting for each category of participants, without the presence of non-participants. Six different moderators and two different observers managed the focus groups. The moderators, who were academics GPs, were experienced, they used the common topic guide with each category of participants and did not take part in the analysis. The two observers were the two researchers who performed the analysis (LL and JSC).

### Analysis

All focus groups were audio-recorded, transcribed and rendered anonymous. Three phases of analysis were performed by the two researchers, in accordance with the Strauss and Corbin grounded theory approach, using NVivo 8 software (QSR International, 2009 Canada). An open coding was first performed on the transcripts of the nine focus groups to reach a consensus definition of the categories and subcategories. An axial coding framework was then developed by the reorganization of the open codes, based on constant comparison and interaction. Finally, selective codes emerged from the prioritization of the axial codes of the four categories of participants. They enabled identification of facilitators and barriers to research in general practice, and of differences and similarities across the four categories of participants.

### Ethics

The North-Western French regional Ethics Committee approved the study (Comité de Protection des Personnes) as an observational study. All participants gave their informed consent.

## Results

Fifty-seven participants were included: 15 patients, 17 non-academic GPs, 14 academic GPs, and 11 other academics (Table 1). The main elements of verbatim for the different categories are presented in Table 2.

**Table 1:** Participant characteristics

***Participant characteristics***	**Patients**	**GPs**	**Academic GPs**	**Other academics**
**(n = 57)**	(n = 15. 2 FG)	(n = 17. 3 FG)	(n = 14. 2 FG)	(n = 11. 2 FG)
**Mean age (range), yrs**	57.9 (39–75)	51.4 (28–62)	46.9 (28–62)	51.7 (34–70)
**Gender**				
Men/women	7/8	16/1	7/7	10/1
**French region (University)**				
North (Lille)	8	7	0	0
Centre-West (Tours)	7	5	7	11
Centre-East (Lyon)	0	5	7	0
**Living/working area**				
Rural	6	7	6	-
Urban	9	10	8	-
**Discipline**				
Medical*	-	-	-	5
Surgery**	-	-	-	1
Other***	-	-	-	5
**Chronic disease**				
Any	7	-	-	-
None	8	-	-	-
**Training practice**				
Yes	-	7	14	-
No	-	10	0	-

*Paediatrics, internal medicine, radiology, haematology, psychiatry.

**Neurosurgery.

***Biology, pharmacology, philosophy, biostatistics, parasitology.

FG = focus groups.

**Table 2:** Responses from the four categories

**Specificity of research in general practice**	AGP 5: “We have a *tutti frutti* compared to other specialties” (FG Lyon)
	AGP 3: “The patient needs to be studied in his natural environment” (FG Tours)
	P12: “Research in general practice should target the prevention of disease, shouldn’t it?” (FG Tours)
	GP 11: “Who caters for patients with diabetes as well as hypertension? We do!” (FG Lyon)
	P8: “I think the relationship between patients and doctors should be taken into account, which is not the case in the specialist fields” (FG Lille)
**Relevance of research in general practice**	GP11: “I see it [research in general practice] as a means to improve the health care provided by doctors, and patients’ health… It is clearly not fundamental research” (FG Lyon)
	GP2: “Although the media influence it a lot, I think research in general practice has helped us to restrict the prescription of antibiotics in viral epidemics, which we had always treated copiously with antibiotics” (FG Lille)
	AGP5: “There is also the improvement of population health. Someone in good health doesn’t cost society much” (FG Lyon)
**Recognition of professionals, patients and the discipline**	AGP10: “I did a study on this subject, the title was something like "being an investigator improves the quality of GP practice” (FG Tours)
	AGP3: “How can we get recognition from other researchers? It is also when people from other fields consider you as a researcher” (FG Lyon)
	OA1: “I think research and publications are the best way to show your real efficacy as a GP, in your field, your domain” (FG Tours) AGP 2: “A speciality without research is not a speciality” (FG Tours)
	GP3: “The first collaborator is the patient…” (FG Lille)
**Pitfalls**	P2a: “[the GP] would have difficulty holding his own amongst specialists and could be seen as an amateur” (FG Lille)
	AGP 10: “Most GPs do not recognize academic GPs and continue to identify themselves “like when they were little” with academic hospital specialists they met during their hospital training, relying only on them to spread the good word” (FG Tours)
	AGP6: “We [academic GPs] are teaching scientific knowledge at the expense of our clinical practice… I feel a kind of a threat in this issue” (FG Lyon)
	P2b: “If someone does research in his speciality, he distances himself from day-to-day practice and from his patients” (FG Lille)
**Feasibility**	P9a: “A GP has contacts with a much wider range of people [than a hospital practitioner]” (FG Tours)
	OA7: “I’ll be pragmatic. Research in general practice is the research done by GPs” (FG Tours)
	OA1: “Research in general practice seems recent to me. My personal point of view is that clinical research in general practice is an innovation” (FG Tours)
	GP13a : “Research to me suggests a particular speciality and technological innovations very far removed from general practice” (FG Tours)
	P9b: “For me, the image of medical research in France is someone everyone knows, Pasteur, who found the rabies vaccine“ (FG Tours)
	P1 : “[GPs] have so many patients that they are not able to do research” (FG Lille)
	AGP3: “We are paid with a fee system, which means time is money, so this system is a major barrier to research” (FG Lyon) GP13b: “For example, it is not in an isolated practice that we can get a sufficient sample of patients to draw general conclusions“ (FG Tours)

### Specificity of research in general practice

Most of the participants from the four participant categories considered the patients consulting in general practice as a specific population, although this specificity covered a range of health problems managed in general practice, from acute benign illnesses and emerging conditions to severe chronic diseases (AGP5). Many participants from the four participant categories considered that studying patients in their natural environment was a characteristic of this research. (AGP3). Research on screening and prevention was seen by some participants in the four categories as an important part of research in general practice (P12). Only some GPs underlined the importance of research on co-morbidities (GP11). Only some GPs and patients identified the communication issue as critical (P8). Some research methods, such as cost-effectiveness, qualitative or mixed methods were regarded as particularly appropriate only by academic GPs and other academics; the other participant categories did not highlight this issue. One non-GP academic insisted on the value of “pragmatic studies”, because of the wide, unselected population attending primary care.

### Relevance of research in general practice

According to some participants in the four categories, research in general practice had a major role to play in health care improvement (GP11). Only some GPs and patients highlighted the usefulness of this type of research for improving patient compliance with care by fostering professional, empathetic and evidence-based management of patients (GP2). Some patients considered that their GP could be more competent in practice if he or she was also a medical researcher. For only some academic and non-academic GPs, one potential objective was to minimize healthcare costs while ensuring quality, while this was not mentioned by the other participant categories (AGP5).

### Recognition of professionals, patients and the discipline

Most GPs and academic GPs pointed out that being an investigator could enhance the quality of health care provided and encourage continuing medical education (AGP10). This was not evoked by the other participant categories. Rewarding the role of the investigators by remuneration was thought appropriate by some GPs and by academic GPs, but not by the other participant categories. Some academic GPs and other academics emphasised the visibility provided by publications, as with any scientific discipline (AGP3, OA1, AGP2). This was not subscribed to by the patients or the GPs. Only two GPs considered that research in general practice was a means to value patients as active collaborators, who might even be paid for their participation (GP3).

### Pitfalls

Most of the participants from the four categories feared that research in general practice might be misused by pharmaceutical industries, especially because pharmaceutical firm representatives meet GPs and in some cases recruit them. Some participants from the four categories perceived rivalries between academic GPs and other academics, who might consider GPs as competing in the university rather negatively (P2a). One academic GP pointed out a possible conflict with other medical specialists for the leadership of primary care research (AGP10). Some GPs and patients stated that the main role of GPs in research was to collect data rather than being involved in study design or management. This restrictive role was seen by some academic GPs as a danger for the conduct of research. Only a few academic GPs were concerned about a possible gap between research and practice, more likely with the integration of general practice departments in the universities. In their opinion, the resulting danger could be the development of university corporatism (AGP6). This was not described by the other participant categories. Only some GPs and patients but not the two academic categories discussed a possible “dehumanisation of practice”, should primary care research lead to neglecting the individual (P2b).

### Feasibility

An asset highlighted by some participants from the four categories was that research in general practice can benefit from GP access to a large, diversified population (P9a), which can improve validity, and form trust-based relationships with patients, facilitating recruitment. Some non-GP academics and academic GPs described the position of the GP in this research as central (OA7).

Some non-GP academics were, however, not aware of the existence of any research in general practice and therefore considered it as a new field (OA1). In particular, they did not know of any international journals of general practice. A lot of participants from the four categories entertained representations of the typical medical researcher as a “laboratory worker”, distant from the clinical practice setting (GP13a, P9b). Most of the participants from the four categories underlined lack of time as a major barrier for research in general practice (P1). According to most participants from the GP, the academic GP and the patient categories, the method of remuneration of French GPs, based on fee for service, had an influence on their low involvement in research (AGP3). Several GPs could not link research to practice and viewed medical research only as fundamental research, unlike academic GPs. The various types of practice, frequently in isolation, were also considered only by some GPs and academic GPs as a limitation for collecting data on a wider scale (GP13b).

While the specific views of patients and non-academic GPs were mostly centred on professional issues, the specific views of academics other than GPs were mostly centred on technical issues. Academic GPs shared both viewpoints, expressing the same expectations as GPs in terms of recognition and remuneration, and as other academics in terms of public health issues. Patient views were quite similar to those of GPs in terms of professional issues. The main subthemes are set out in Table 3, according to the four participant categories and to professional or technical issues.

**Table 3:** Subthemes subsumed in the five main themes, according to the four participant categories

	**General practitioners**	**Patients**	**Academic GPs**	**Other academics**
Specificity	**Specific population**			
	**Natural environment**			
	**Screening and prevention**			
	Patient-physician communication	Patient-physician communication	*Diversified research methods*	*Diversified research methods*
Relevance	**Health care improvement**			
	Patient compliance	Patient compliance	Health care cost minimization	
	Health care cost minimization			
Recognition	GPs as investigators		GPs as investigators	*Publications*
	Patients as active collaborators		*Publications*	
Pitfalls	**GPs misused by pharmaceutical industries**			
	**Perceived rivalries in the university**			
	GPs just collecting data	GPs just collecting data	*University corporatism*	
	Dehumanisation of practice	Dehumanisation of practice		
Feasibility	**Access to a diversified population**			
	**Typical medical researcher as a “laboratory worker”**			
	**Lack of time**			
	**Access to a diversified population**			
	Fee for service system	Fee for service system	Fee for service system	
	Isolation of practice		Isolation of practice	

**Bold** characters denote common subthemes between the four participant categories.

Normal characters denote specific subthemes related to professional issues.

*Italics* denote specific subthemes related to technical issues.

## Discussion

An emerging theory built from our data shows a tension between the views and of the four participant categories. Academic GPs seem to be an interface between other academics, centred on technical issues of primary care research, on one hand, and patients and non-academic GPs, centred on professional issues, on the other hand. This university corps of GPs could play a key role in resolving conflicts between the views of the other actors involved, bridging universities and general practices. According to the verbatim derived from GPs, academic GPs, other academics and patients, the perceived legitimacy of research in general practice was improving overall in France. This type of research was perceived as deeply rooted in day-to-day practice, with specific advantages and drawbacks. Solutions for overcoming the barriers also emerged from the analysis.

### Improving legitimacy

The legitimacy of research in general practice was not disputed by the participants. Specific fields, such as prevention, practice evaluation, or communication, and specific methods such as pragmatic studies, mixed methods designs or cost-effectiveness studies, were described by the French stakeholders in accordance with international position papers [12]. Because of its social, environmental and psychological perspectives, research in general practice seems closer to patients’ different needs and concerns than other fields of medical research. In addition, this research could have a structuring role for the quality of primary care, and beyond this, for public health at large [3]. As reported by academic GPs in our study, the combination of quantitative and qualitative methods is particularly relevant for studying the complex clinical situations managed in primary care medicine worldwide [13]. The development of skills in these complementary research methods has contributed to the emergent recognition of general practice as an academic discipline in French university [14]. In our study, academic GPs recognized their possible role as leaders in the research process while other academics were more equivocal, whereas GPs and patients mainly thought the GP had only a recruitment role. These differences of view show that French academic GPs need to reinforce their legitimacy in conducting research studies. The university setting offers opportunities for collaboration with researchers from other disciplines, which can reinforce the legitimacy of research in general practice, as it has done in other countries [15]. French academic GPs need to prove the added value of research in general practice, in addition to facilitating data collection in general practice [2]. Multidisciplinary collaboration in research should find ways to develop fair partnerships in the highly competitive environment in the universities. The short-sighted view of the medical researcher as a laboratory worker is a barrier to developing research in general practice in France. It may result from the priority given in France to fundamental research [16].

### Grounding in day-to-day practice

GP researchers are also clinicians and relevant research questions often emerge from routine practice [17]. General practice is diversified, and various issues with a poor evidence base need to be studied, including clinical conditions, patient behaviours and the organisation of health care [18]. The population managed by GPs is very large, as the participants from the four categories mentioned, and representative groups of patients can be recruited according to research aims. The recruitment process can be facilitated by the trust relationships established with patients over time [19]. Unlike hospital-based research, research in the general practice setting enables various research designs, including observational and pragmatic interventional studies. These studies can foster the development of guidelines that are more relevant to situations encountered in primary care [20].

However, GPs reported being usually overwhelmed by daily clinical duties and felt they could not be available for research, as reported in previous studies [21]. Studies in primary care can be administratively demanding, resulting in poor-quality studies [22]. According to our findings, the fee for service system prevailing in France is probably not an incentive for GPs to participate in research, especially for those practicing in isolation. The payment system has already been identified as a barrier for developing research [23]. French academic GPs currently face the new challenging institutional requirement of the triple task combining teaching, research and patient care [9]. Thus academic duties may keep them away from their practice, and create a hiatus between research and clinical work, as already observed in Germany [22].

### Overcoming the barriers

Regional or national research networks are often considered as a key to developing research in primary care [24]. These networks should ideally be attached to universities, in order to provide scientific input [25]. Such networks, which exist in several European countries but not yet in France, could be a solution to limit the possible gap between research and practice [26]. The French healthcare system should implement this type of organization, already established and successful in the UK and in the Netherlands. Basing research questions on GPs’ expectations in a bottom-up approach can improve their participation in research projects [27]. Consulting patients on research projects using a community approach can facilitate their recruitment, as experienced in an Australian primary care research network. In this model, healthcare users are asked to participate in primary care networks to elaborate action-research projects [28]. GPs should get regular, specific training on patient recruitment and data collection, in order to improve their involvement and motivation [29]. Their practices should be computerized, using interoperable software able to generate standardized data from electronic health records [30]. Unlike the United Kingdom, where GPs use three main interoperable types of software, more than 15 different systems, weakly structured and poorly interoperable, are available in France. This heterogeneity represents a key barrier for the development of efficient research networks [31,32]. Multi-professional health centres with shared information systems could provide opportunities for collaborative research in primary care networks [17]. Institutional and public funding should also be available for specific research projects, especially to supply networks with research assistants [33]. In all countries, the publication of findings from general practice research is a priority to improve its perceived legitimacy and alter social representations [34]. Reporting of research findings to the investigators is important to reinforce their motivation. They can also be communicated to patients as suggested in an American study [35].

The means for developing research capacities are fairly similar across Western countries [3]. But the implementation of these means has varied considerably, especially in Europe [36]. The United Kingdom, the Scandinavian countries, the Netherlands and the USA are ahead, as they have been building primary care research networks for the last three decades [37]. In contrast, France and Germany have extended their research capacity more slowly, and GPs from Southern and Eastern European countries still have to struggle for the recognition of their discipline, before developing primary care research [36]. France has several assets, in particular an emerging university corps in general practice and a large GP workforce [38].

A recent report to the French Minister of Health and proposals from the General Practice/INSERM (National institute for medical research and health) Interface Commission described solutions to overcome barriers to research in general practice, including creating research networks, providing specific funding and training investigators [39,40].

### Strengths and weaknesses

We checked that our study design conformed to 30 out of the 32 criteria of the COREQ checklist for reporting qualitative research [41]. The two criteria not-validated concerned the feedback to participants on the transcripts and findings. Several qualitative and quantitative studies have previously explored perceptions of general practice research [18,21,22,29]. They were limited in terms of participant categories (only GPs, or even only academic GPs) [18,22], or were restricted to a specific clinical domain [29]. To our knowledge, this study is the first to explore the views of patients and various academics. Patient views were important to collect because they provide insight into the feasibility of their recruitment in primary care studies. Views from the academics in other disciplines are also relevant because academic GPs are hoping for recognition from, and collaboration with, their peers in the university. The fact that focus groups were conducted in 2010 could be a potential limitation of our study. However, the main assets and barriers in the French primary health care system have not dramatically changed, and the solutions proposed by the Health Authorities and GP organisations still remain to be implemented. Women were under-represented in the GP and the other academic categories. In France, the proportion of female GPs is around 40% [38]. Most female GPs contacted declined to participate in the focus groups because they were scheduled in the evening. However, according to a French study, GP gender has little influence on the motivation to participate in research [10]. Women represented a small proportion of the other academics, presumably because they represent only 20% of the professors in the universities, and there was indeed no refusal to participate from this group [42].

We presumably reached overall data saturation across the participant categories and possibly within each category, as the last focus group for each category did not provide any substantially new information.

## Conclusion

Academics GPs are in a position to play a role of interface between the universities and general practices. Although the role of GPs is perceived differently by different protagonists, research in general practice has an undisputed legitimacy in France. Its grounding in day-to-day practice enables pragmatic approaches but also presents several drawbacks. Solutions for overcoming the barriers include research networks with appropriate resources and training and scientifically sound collaborative research projects, as already implemented in leading countries.

## Competing interests

The authors declare that they have no competing interests.

## Authors’ contributions

JSC and LL had the idea for the study, obtained funding, planned all focus groups and moderated some of the focus group sessions, recruited other moderators, participated as observers, directed the design and analysis of the study, wrote the first draft of the paper, and are the guarantors. JPL conducted some of the focus groups, transcribed some of the verbatim. JSC, LL, JPL, EL contributed to the qualitative analysis, data interpretation, and writing of the article. All authors read and approved the final manuscript.

## Pre-publication history

The pre-publication history for this paper can be accessed here:

http://www.biomedcentral.com/1471-2296/15/136/prepub
